# 3,5-Dichloro­salicylaldehyde

**DOI:** 10.1107/S1600536808011021

**Published:** 2008-04-26

**Authors:** Isha Azizul, Seik Weng Ng

**Affiliations:** a Department of Chemistry, University of Malaya, 50603 Kuala Lumpur, Malaysia

## Abstract

The title compound (systematic name: 3,5-dichloro-2-hydroxy­benzaldehyde), C_7_H_4_Cl_2_O_2_, crystallizes as discrete mol­ecules, the conformation of which may be influenced by an intra­molecular hydr­oxy–carbonyl O—H⋯O hydrogen bond.

## Related literature

For the crystal structure of 3′,5′-dichloro­acetophenone, see: Filarowski *et al.* (2004 ▶).
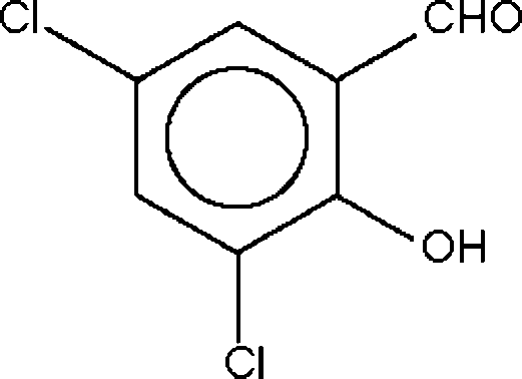


## Experimental

### 

#### Crystal data

C_7_H_4_Cl_2_O_2_
*M* *_r_* = 191.00
Monoclinic, 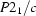

*a* = 8.2823 (2) Å
*b* = 13.7412 (3) Å
*c* = 7.0973 (2) Å
β = 115.185 (2)°
*V* = 730.95 (3) Å^3^
*Z* = 4
Mo *K*α radiation
μ = 0.82 mm^−1^
*T* = 100 (2) K
0.25 × 0.15 × 0.05 mm

#### Data collection

Bruker SMART APEX diffractometer
Absorption correction: multi-scan (*SADABS*; Sheldrick, 1996 ▶) *T*_min_ = 0.701, *T*_max_ = 0.960
8436 measured reflections
1672 independent reflections
1303 reflections with *I* > 2σ(*I*)
*R*_int_ = 0.058

#### Refinement

*R*[*F* ^2^ > 2σ(*F* ^2^)] = 0.037
*wR*(*F* ^2^) = 0.101
*S* = 1.05
1672 reflections
104 parameters
1 restraint
H atoms treated by a mixture of independent and constrained refinement
Δρ_max_ = 0.59 e Å^−3^
Δρ_min_ = −0.49 e Å^−3^

### 

Data collection: *APEX2* (Bruker, 2007 ▶); cell refinement: *SAINT* (Bruker, 2007 ▶); data reduction: *SAINT*; program(s) used to solve structure: *SHELXS97* (Sheldrick, 2008 ▶); program(s) used to refine structure: *SHELXL97* (Sheldrick, 2008 ▶); molecular graphics: *X-SEED* (Barbour, 2001 ▶); software used to prepare material for publication: *publCIF* (Westrip, 2008 ▶).

## Supplementary Material

Crystal structure: contains datablocks global, I. DOI: 10.1107/S1600536808011021/lh2606sup1.cif

Structure factors: contains datablocks I. DOI: 10.1107/S1600536808011021/lh2606Isup2.hkl

Additional supplementary materials:  crystallographic information; 3D view; checkCIF report

## Figures and Tables

**Table 1:** Hydrogen-bond geometry (Å, °)

*D*—H⋯*A*	*D*—H	H⋯*A*	*D*⋯*A*	*D*—H⋯*A*
O1—H1⋯O2	0.84 (1)	1.87 (2)	2.628 (3)	149 (3)

**Fig. 1.**
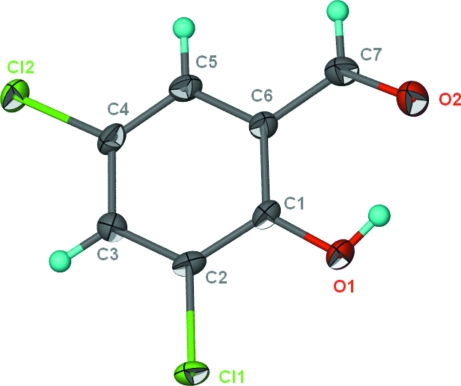
70% Probability thermal ellipsoid plot of 3,5-dichlorosalicylaldehyde. Hydrogen atoms are drawn as spheres of arbitrary radius.

## supplementary crystallographic information

### Comment

The intramolecular hydrogen bonds in small molecules such as *ortho*-hydroxyacetopheonone and its derivatives has been extensively studied, both theoretically and crystallographically. Such compounds can exist in a keto-enol equilibrium. For 3',5'-dichloroacetophenone, geometry-optimization calculations suggest that the presence of two chlorine substituents raises the acidity of the hydroxyl proton and decreases the basicity of the carbonyl function. The O···O distance in the hydrogen bond is 2.567 (3) Å (Filarowski *et al.*, 2004).

The hydrogen bond in the title molecule (I) is longer with an O···O distance of 2.628 (3) Å. 3,5-Dichlorosalicylaldehyde (I) exists as a monomeric compound (Fig. 1); the molecule is flat and all bond dimensions are normal.

### Experimental

The compound was purchased from Aldrich Chemical Company; the chemical exists as colorless prismatic crystals. The bulk chemical has a yellow color.

### Refinement

Carbon-bound H-atoms were placed in calculated positions (C—H 0.95 Å) and were included in the refinement in the riding model approximation, with *U*(H) set to 1.2*U*(C). The oxygen-bound H atom was located in a difference Fourier map, and was refined with a distance restraint of O–H 0.84±0.01 Å; its temperature factor was freely refined.

### Crystal data

**Table:**

C_7_H_4_Cl_2_O_2_	*F*(000) = 384
*M**_r_* = 191.00	*D*_x_ = 1.736 Mg m^−^^3^
Monoclinic, *P*2_1_/*c*	Mo *K*α radiation, λ = 0.71073 Å
Hall symbol: -P 2ybc	Cell parameters from 3128 reflections
*a* = 8.2823 (2) Å	θ = 3.0–28.2°
*b* = 13.7412 (3) Å	µ = 0.82 mm^−^^1^
*c* = 7.0973 (2) Å	*T* = 100 K
β = 115.185 (2)°	Block, colorless
*V* = 730.95 (3) Å^3^	0.25 × 0.15 × 0.05 mm
*Z* = 4	

### Data collection

**Table:**

Bruker SMART APEXII diffractometer	1672 independent reflections
Radiation source: fine-focus sealed tube	1303 reflections with *I* > 2σ(*I*)
graphite	*R*_int_ = 0.058
ω scans	θ_max_ = 27.5°, θ_min_ = 2.7°
Absorption correction: multi-scan (*SADABS*; Sheldrick, 1996)	*h* = −10→10
*T*_min_ = 0.701, *T*_max_ = 0.960	*k* = −17→17
8436 measured reflections	*l* = −9→9

### Refinement

**Table:**

Refinement on *F*^2^	Primary atom site location: structure-invariant direct methods
Least-squares matrix: full	Secondary atom site location: difference Fourier map
*R*[*F*^2^ > 2σ(*F*^2^)] = 0.037	Hydrogen site location: inferred from neighbouring sites
*wR*(*F*^2^) = 0.101	H atoms treated by a mixture of independent and constrained refinement
*S* = 1.05	*w* = 1/[σ^2^(*F*_o_^2^) + (0.0416*P*)^2^ + 0.8939*P*] where *P* = (*F*_o_^2^ + 2*F*_c_^2^)/3
1672 reflections	(Δ/σ)_max_ = 0.001
104 parameters	Δρ_max_ = 0.59 e Å^−^^3^
1 restraint	Δρ_min_ = −0.49 e Å^−^^3^

### Fractional atomic coordinates and isotropic or equivalent isotropic displacement parameters (Å^2^)

**Table:**

	*x*	*y*	*z*	*U*_iso_*/*U*_eq_	
Cl1	0.17794 (9)	0.14230 (4)	0.48280 (10)	0.01972 (18)	
Cl2	0.15345 (9)	0.53148 (4)	0.39904 (11)	0.02166 (19)	
O1	0.5328 (3)	0.17615 (13)	0.8080 (3)	0.0200 (4)	
H1	0.631 (3)	0.192 (3)	0.905 (4)	0.038 (10)*	
O2	0.7916 (3)	0.28892 (14)	1.0580 (3)	0.0252 (4)	
C1	0.4488 (3)	0.25960 (17)	0.7232 (4)	0.0155 (5)	
C2	0.2766 (3)	0.25497 (17)	0.5629 (4)	0.0164 (5)	
C3	0.1851 (4)	0.33804 (17)	0.4659 (4)	0.0168 (5)	
H3	0.0680	0.3339	0.3572	0.020*	
C4	0.2680 (4)	0.42815 (17)	0.5306 (4)	0.0177 (5)	
C5	0.4355 (4)	0.43592 (17)	0.6906 (4)	0.0179 (5)	
H5	0.4890	0.4980	0.7336	0.021*	
C6	0.5266 (3)	0.35155 (17)	0.7897 (4)	0.0160 (5)	
C7	0.7028 (4)	0.35892 (18)	0.9634 (4)	0.0206 (6)	
H7	0.7517	0.4220	1.0058	0.025*	

### Atomic displacement parameters (Å^2^)

**Table:**

	*U*^11^	*U*^22^	*U*^33^	*U*^12^	*U*^13^	*U*^23^
Cl1	0.0225 (3)	0.0110 (3)	0.0228 (3)	−0.0036 (2)	0.0068 (3)	−0.0026 (2)
Cl2	0.0204 (3)	0.0122 (3)	0.0276 (4)	0.0029 (2)	0.0056 (3)	0.0051 (2)
O1	0.0193 (10)	0.0111 (8)	0.0243 (11)	0.0025 (7)	0.0042 (9)	0.0025 (7)
O2	0.0211 (10)	0.0197 (9)	0.0272 (11)	0.0004 (8)	0.0030 (9)	0.0033 (8)
C1	0.0182 (13)	0.0111 (10)	0.0180 (12)	0.0028 (9)	0.0083 (11)	0.0016 (9)
C2	0.0216 (13)	0.0106 (10)	0.0186 (12)	−0.0017 (9)	0.0100 (11)	−0.0016 (9)
C3	0.0163 (13)	0.0162 (12)	0.0172 (13)	−0.0004 (9)	0.0063 (11)	−0.0007 (9)
C4	0.0201 (14)	0.0114 (11)	0.0213 (13)	0.0038 (9)	0.0086 (12)	0.0032 (9)
C5	0.0202 (14)	0.0108 (11)	0.0228 (14)	−0.0024 (9)	0.0093 (12)	0.0000 (9)
C6	0.0151 (13)	0.0123 (11)	0.0195 (13)	−0.0004 (9)	0.0064 (11)	0.0003 (9)
C7	0.0212 (14)	0.0149 (12)	0.0229 (14)	−0.0029 (10)	0.0068 (12)	−0.0003 (10)

### Geometric parameters (Å, °)

**Table:**

Cl1—C2	1.730 (2)	C3—C4	1.395 (3)
Cl2—C4	1.742 (2)	C3—H3	0.9500
O1—C1	1.343 (3)	C4—C5	1.373 (4)
O1—H1	0.840 (10)	C5—C6	1.399 (3)
O2—C7	1.223 (3)	C5—H5	0.9500
C1—C2	1.397 (4)	C6—C7	1.459 (4)
C1—C6	1.406 (3)	C7—H7	0.9500
C2—C3	1.381 (3)		
			
C1—O1—H1	107 (3)	C5—C4—Cl2	120.53 (19)
O1—C1—C2	118.7 (2)	C3—C4—Cl2	117.9 (2)
O1—C1—C6	122.7 (2)	C4—C5—C6	119.4 (2)
C2—C1—C6	118.5 (2)	C4—C5—H5	120.3
C3—C2—C1	121.5 (2)	C6—C5—H5	120.3
C3—C2—Cl1	119.6 (2)	C5—C6—C1	120.3 (2)
C1—C2—Cl1	118.96 (18)	C5—C6—C7	119.9 (2)
C2—C3—C4	118.7 (2)	C1—C6—C7	119.8 (2)
C2—C3—H3	120.6	O2—C7—C6	124.1 (2)
C4—C3—H3	120.6	O2—C7—H7	118.0
C5—C4—C3	121.6 (2)	C6—C7—H7	118.0
			
O1—C1—C2—C3	−178.3 (2)	Cl2—C4—C5—C6	−178.17 (19)
C6—C1—C2—C3	2.0 (4)	C4—C5—C6—C1	1.3 (4)
O1—C1—C2—Cl1	0.8 (3)	C4—C5—C6—C7	−178.4 (2)
C6—C1—C2—Cl1	−178.90 (18)	O1—C1—C6—C5	177.7 (2)
C1—C2—C3—C4	0.1 (4)	C2—C1—C6—C5	−2.7 (4)
Cl1—C2—C3—C4	−179.03 (19)	O1—C1—C6—C7	−2.6 (4)
C2—C3—C4—C5	−1.5 (4)	C2—C1—C6—C7	177.1 (2)
C2—C3—C4—Cl2	177.52 (18)	C5—C6—C7—O2	−179.8 (3)
C3—C4—C5—C6	0.9 (4)	C1—C6—C7—O2	0.5 (4)

### Hydrogen-bond geometry (Å, °)

**Table:**

*D*—H···*A*	*D*—H	H···*A*	*D*···*A*	*D*—H···*A*
O1—H1···O2	0.84 (1)	1.87 (2)	2.628 (3)	149 (3)

## References

[bb1] Barbour, L. J. (2001). *J. Supramol. Chem.* **1**, 189–191.

[bb2] Bruker (2007). *APEX2* and *SAINT* Bruker AXS Inc., Madison, Wisconsin, USA.

[bb3] Filarowski, A., Koll, A., Kochel, A., Kalenik, J. & Hansen, P. E. (2004). *J. Mol. Struct.* **700**, 67–72.

[bb4] Sheldrick, G. M. (1996). *SADABS* University of Göttingen, Germany.

[bb5] Sheldrick, G. M. (2008). *Acta Cryst.* A**64**, 112–122. 10.1107/S0108767307043930 18156677

[bb6] Westrip, S. P. (2008). *publCIF* In preparation.

